# Identifying Adverse Effects of HIV Drug Treatment and Associated Sentiments Using Twitter

**DOI:** 10.2196/publichealth.4488

**Published:** 2015-07-27

**Authors:** Cosme Adrover, Todd Bodnar, Zhuojie Huang, Amalio Telenti, Marcel Salathé

**Affiliations:** ^1^ Center for Infectious Disease Dynamics Department of Biology Penn State University University Park, PA United States; ^2^ J. Craig Venter Institute La Jolla, CA United States

**Keywords:** Twitter, HIV, AIDS, pharmacovigilance, mTurk, mechanical Turk

## Abstract

**Background:**

Social media platforms are increasingly seen as a source of data on a wide range of health issues. Twitter is of particular interest for public health surveillance because of its public nature. However, the very public nature of social media platforms such as Twitter may act as a barrier to public health surveillance, as people may be reluctant to publicly disclose information about their health. This is of particular concern in the context of diseases that are associated with a certain degree of stigma, such as HIV/AIDS.

**Objective:**

The objective of the study is to assess whether adverse effects of HIV drug treatment and associated sentiments can be determined using publicly available data from social media.

**Methods:**

We describe a combined approach of machine learning and crowdsourced human assessment to identify adverse effects of HIV drug treatment solely on individual reports posted publicly on Twitter. Starting from a large dataset of 40 million tweets collected over three years, we identify a very small subset (1642; 0.004%) of individual reports describing personal experiences with HIV drug treatment.

**Results:**

Despite the small size of the extracted final dataset, the summary representation of adverse effects attributed to specific drugs, or drug combinations, accurately captures well-recognized toxicities. In addition, the data allowed us to discriminate across specific drug compounds, to identify preferred drugs over time, and to capture novel events such as the availability of preexposure prophylaxis.

**Conclusions:**

The effect of limited data sharing due to the public nature of the data can be partially offset by the large number of people sharing data in the first place, an observation that may play a key role in digital epidemiology in general.

## Introduction

### The Sharing of Health Information on Twitter

Twitter is a popular microblogging platform where users publicly share information, including personal thoughts and emotions. Everyday, hundreds of millions of tweets are posted on Twitter. This offers a large potential source of information for public health purposes. The sharing of information about personal health is now widely recognized to be a broad phenomenon that occurs in almost any field imaginable [1]. Examples include the sharing of vaccination behavior [2], the sharing of marijuana consumption [3], the sharing of weight loss attempts [4], or the sharing of suicidal thoughts [5]. Moreover, this large pool of available data can be used to estimate the most common side effects of certain pharmaceutical products [6,7]. In this study, we will focus on identifying human immunodeficiency virus (HIV)-infected individuals currently undergoing drug treatment, and on their personal experiences, specifically, with regard to drug toxicities.

We chose a specific question that a priori seemed difficult: to identify infected individuals that would communicate about their HIV status, and more specifically, about their treatment. Drug adverse effects have been a source of concern, both for the medical establishment, and for HIV-infected individuals, due to their prevalence [8,9], and because treatments have to be taken for life. There is a general agreement that toxicity can also affect treatment adherence. Newer agents are increasingly associated with lesser rates and severity of adverse drug effects; however, the user community remains highly sensitive to this central aspect of treatment that influences the quality of life. Information and beliefs can spread rapidly, and influential voices and vehicles of opinion may alter the perception of the community on treatment adequacy.

### Filtering Tweets for the Study

The study that we present is based upon tweets filtered by specific keywords related to HIV and HIV treatments. Our work builds on a growing body of literature that uses combined human and computational approaches to assess health and disease dynamics from digital media [2,10]. Our goal was to determine if there are common adverse effects related to a particular HIV treatment, and to establish overall user sentiment (a positive, neutral, or negative perception) associated with the content of the tweet. Through our study, we defined methods to identify populations of interest. We used crowdsourcing (Amazon Mechanical Turk) to rate tweets to create training samples for our machine learning algorithms. On the analytical side, these algorithms were used to identify most of the tweets posted by our identified community (hereafter, referred to as “signal”): users with HIV whose tweets included references to treatments, symptomatic descriptions, opinions, and feelings. The final filtering of identified tweets was again obtained through crowdsourcing. Despite the limited number of tweets and individuals that may communicate publicly about HIV treatment experiences, we found a surprisingly large proportion of reports of drug toxicity, a high level of precision on drug-specific effects, and a general negative perception of treatment. We believe that monitoring of social media will be informative for the field, and broadly applicable to the surveillance of other therapies with long duration of use, even for those targeted at diseases that are associated with considerable stigma.

## Methods

### Dataset

For this study, we purchased 39,988,306 tweets posted between September 2010 and August 2013. We purchased the data from Gnip Inc, an official Twitter data reseller, which has recently been acquired by Twitter Inc. These tweets represent the full and unbiased stream of data during that period of time. A tweet was included in the dataset if at least one of the following keywords was contained within the tweet: *Sustiva*, *Stocrin*, *Viread*, *FTC*, *Ziagen*, *3TC*, *Epivir*, *Retrovir*, *Viramune*, *Edurant*, *Prezista*, *Reyataz*, *Norvir*, *Kaletra*, *Isentress*, *Tivicay*, *Atripla*, *Trizivir*, *Truvada*, *Combivir*, *Kivexa*, *Epzicom*, *Complera*, *Stribild*, *HIV treatment*, *HIV drug*, *anti-HIV*, *triple therapy HIV*, or *anti HIV*. These keywords were chosen in line with the listing of main antiretroviral agents in use [11]. *AZT* was dropped because of its extreme noise to signal ratio (as *azt* is one of the most used words in Hungarian). For technical reasons, the search query had to be limited in the numbers of search terms used.

### Processing of Tweets

For each collected tweet, we created a list of all the distinct tokens that the tweet contained. If at least one keyword matched at least one item in the list, we kept that tweet. This step reduced our data sample to about 1.8 million tweets, mainly due to the presence of compound words such as *giftcard*, triggered by the keyword *FTC*, which represented a large source of noise. Moreover, a subsample of tweets containing the keyword *FTC* presented a large bias toward information related to the Federal Trade Commission. We decided to discard this subsample from the data.

Hereafter, “signal” denotes our identified tweets posted by patients, and “noise” denotes tweets about unrelated topics, such as objective sentences concerning new discoveries or recent information about HIV in general.

**Figure 1:**
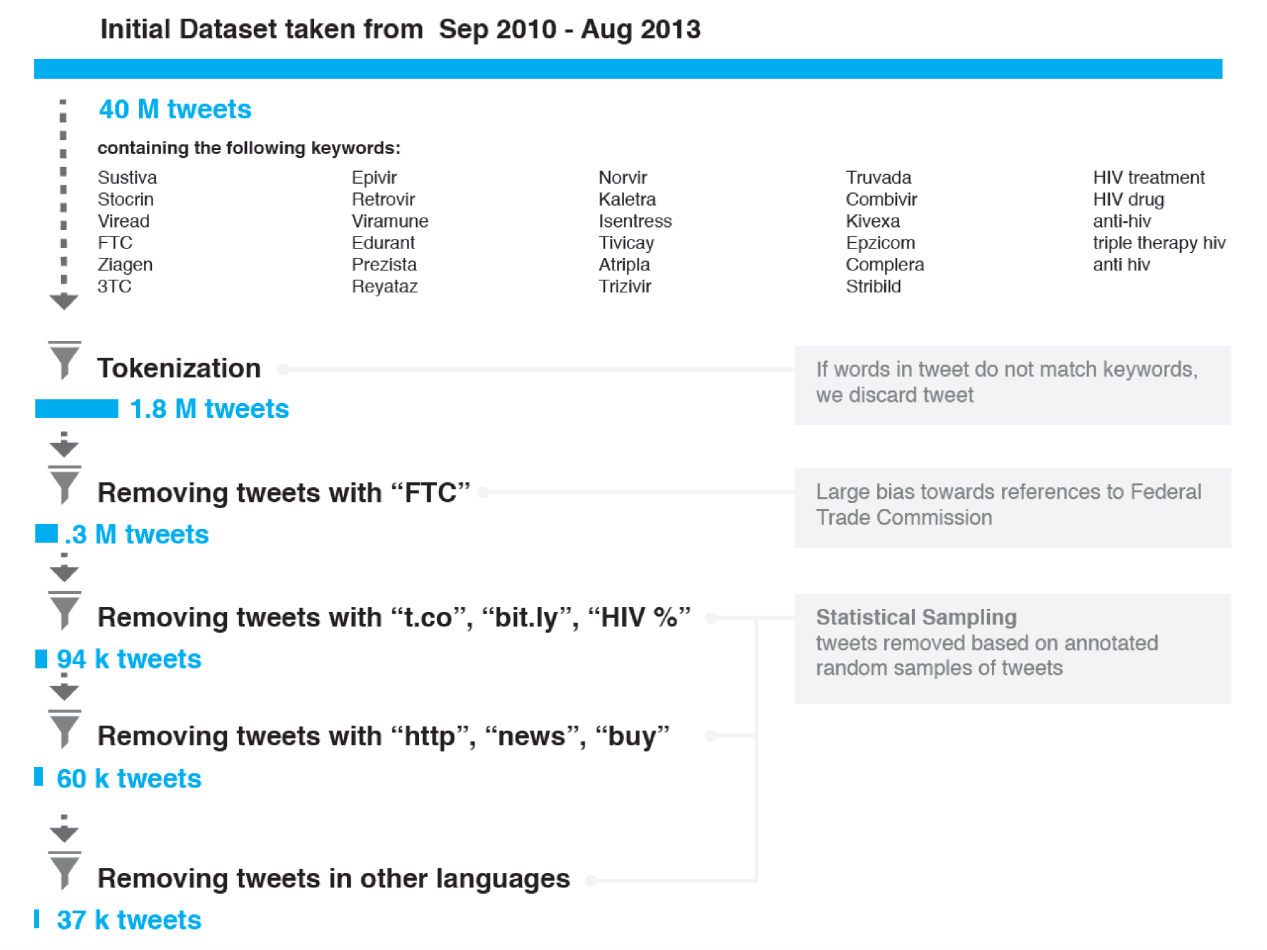
Overview of the different filters applied for the processing of tweets. M=million; k=thousand.

Our statistical sampling analysis (Figure 1 shows this) started with loose criteria to reduce the noise in our sample. We used computational algorithms to randomly select different sets of tweets that contained words from potential noisy sources and sets of tweets that did not contain these “noisy” words. These sets of tweets were manually annotated by two of the authors (CA and TB) as either noise or signal. In the case of a slight indication of subjectivity, the tweet was annotated as signal. If the differences between the two types of sets (ie, containing or not containing specific noisy words) were significant in terms of signal content, we discarded tweets from our dataset that contained those noisy words (for more details, see below under the subheading “Identification of Signal Through Consecutive Filters”). Although we expected to discard some signal tweets through this cleaning process, it provided robustness to our procedure, as it allowed us to increase the percentage of signal tweets in the remaining sample. This higher purity of signal allowed a higher number of crowdsourced signal tweets to define training samples.

### Identification of the Community

The goal of the data curation that we pursued was to purify the original noisy sample of tweets to a sample containing only signal. To do this, we defined a set of features that transformed a tweet into quantitative information (ie, number of words in the tweet, number of adverbs, etc). Moreover, these features were selected based on their separation power between objective information and subjective sentences charged with personal references. In a further step, we combined these features into a single output through a machine learning algorithm.

### Training Samples

To define training samples for the classes of signal, noise, and non-English, we performed a crowdsourcing request of 4000 tweets. There were two Amazon Mechanical Turk workers that rated these tweets. We asked the two workers whether they believed the tweet fulfilled one of the following four criteria: (1) talking about personal medication; (2) talking about medication, but not personally; (3) talking about completely irrelevant topics to our study; and (4) tweet not in English. If both workers agreed on their respective answers concerning a given tweet, we kept the tweet for our analysis. Tweets rated as category (1) were used as signal, and categories (2) and (3) as noise. We had an agreement of about (3118/4000) 77.95% between workers that rated our tweets. Moreover, we used tweets rated as non-English as a control sample in order to remove foreign language tweets. We removed non-English tweets with a method described in see Multimedia Appendix 1.

### Machine Learning Classifier

We utilized the Toolkit for Multivariate Analysis library [12] to define our machine learning classifier. We computed the signal efficiency versus noise rejection for four types of classifiers: (1) Boosted Decision Trees with AdaBoost (BDT), (2) Support Vector Machines (SVM), (3) Boosted Decision Trees with Bagging (BDTG), and (4) artificial neural networks. The figure of merit that we used to optimize our classifier was the noise rejection efficiency at a signal efficiency of 90%. We used this point on the receiver operating characteristic curve because it represents an optimal threshold to keep a large fraction of relevant signal tweets, while removing noise at a level that permits a final human validation. This further crowdsourcing was the final step in our identification of signal and was applied right after the machine learning algorithm.

### Annotation of Side Effects and Sentiment Scores

An important aspect of analysis of Twitter data that cannot easily be obtained through other digital media (eg, search queries from Google or Bing) is the possibility to attribute an overall “sentiment”. For this study, we hand-rated tweets on a sentiment scale ranging from -5 to 5, in steps of 1. The former indicates an extremely negative sentiment and the latter an extremely positive sentiment. The following tweets are examples from the final dataset.

5: Hey guys I m officially undetectable!!!! Take that #hiv! CD4 went up bout 150 pts also! yaya!! #atripla

4: Whoever invented Atripla (and it s component parts - it s three drugs in one) are geniuses. I love you.

3: and more exciting and totally separate from this weekend, I am moving drug combinations from Atripla.

2: I hear Truvada makes you fat like a Tellytubby. I am soooooo excited to finally be able to look like my hero, Tinky Winky!

1: @TheBodyDotCom Taking Good food #nutrition as part of myother side effects.

0: So,you better all get started taking Truvada (like I already do). It s OK, coz the FDA say so. hmmmm

-1: #Atripla vs #HIV These meds seem to be getting short andwas still flying.

-2: Atripla is a B**CH when you have to be up early in thefor 6 hours!

-3: Oh boy here goes that feeling I hate.... #Atripla

-4: I think I ll pass on the Atripla again today though. I feel weak but my future is so bleak I just want to waste away.

(There were no tweets that we felt met out criteria of an extremely negative -5 rating.) Our measure of sentiment, Ψ, is the average of the sentiments of all tweets in a given time window, after having assigned a systematic uncertainty of 1 to each rating, which lead to a total uncertainty in the average of 1 / √ N. The sample of pure signal tweets was annotated by one of the authors (CA). Side effects were transcribed with no previous knowledge of drug toxicities.

### Identification of Signal Through Consecutive Filters

After we applied the cleaning process described above, our dataset contained a larger fraction of possible signal tweets. In this section, we detail the steps taken to reach a sample that contained only signal tweets (Figure 1).

In a first step, we selected three random samples of 500 tweets containing tokens *t.co, bit.ly*, and starting with the term *HIV*. In Twitter, the tokens *t.co* and *bit.ly* are used as part of hyperlinks. We selected also one random sample of 500 tweets not containing these words. For the three filters, we found 0, 1, and 1 possible signal tweets in the first samples, respectively. There were 24 possible signal tweets that were found in the last sample where the tokens *t.co*, *bit.ly*, and starting with *HIV* were excluded. From this, we derived that 7.4 ± 2.7 possible signal tweets were expected to be found in 500 tweets taken from the original 316,081. The subsample with *t.co, bit.ly,* and starting with *HIV* contained a very low fraction of possible signal tweets, and was discarded from the dataset. Our findings indicate that these tokens appear within tweets addressing an objective idea.

In a second step, we checked tweets containing *http*, *news*, or *buy*. We selected three random samples of 140 tweets and found zero tweets in each of the three cases. As we found 24 possible signal tweets in a total of 500 tweets, we expected 6.7 ± 2.6 possible signal tweets in 140 tweets, assuming a Poisson distribution. We computed the probability to discard a possible signal tweet if we removed tweets containing *http*, *news*, or *buy* to be 4 × 10^-6^. After we discarded tweets containing *http*, *news*, or *buy*, our dataset was reduced substantially.

In the final step, we separated two samples of 150 tweets containing *free*, *buy*, *de*, *e*, *za*, *que*, *en*, *lek*, *la*, *obat*, *da*, *majka*, *molim*, *hitno*, *mil*, or *africa*. These tokens were selected on the basis of forming part of foreign dictionaries, belonging to retailing companies selling their products, or seemingly forming part of news. We found zero possible signal tweets in both cases. Then, we estimated that the probability of losing possible signal tweets was less than 5 × 10^-6^, if we removed tweets containing at least one of this large set of words. Following the aforementioned processing of tweets, the sample was further reduced to 37,337 tweets, or about 0.933% of the original sample size of 40 million tweets. Figure 1 presents a visualization of the various filters applied to our dataset in the cleaning process.

To identify tweets posted by our community of interest, we continued to filter the sample of 37,337 tweets. An SVM classifier, trained using the variables (see Multimedia Appendix 1) personalcount, tagnoun, sis noise, sis signal, bigrams noise, is english, common noise, common signal, and ncharacters, allowed to reduce the noise of the aforementioned sample by (481/603) 79.8%, while keeping (26/30) 87% of the signal tweets (see Multimedia Appendix 1, Figure S1). We were confident with the performances of our classifier, as the results obtained using a different testing (cross-check) sample agree within the statistical uncertainties. The classifier used was trained using 603 noise and 49 signal tweets, respectively. The performances of this classifier were tested and then validated using two different sample sets that contained each 603 noise and 30 signal tweets, respectively.

The output of our classifier is a real number between 0 and 1. Higher values indicate higher probability of being signal. In order to estimate the threshold to apply to our sample, we used the annotated signal tweets and computed the 90% signal efficiency threshold to be 0.45. Therefore, we parsed our entire sample of tweets through our classifier and kept only those tweets with classifier outputs larger than 0.45. As a result of this filtering, our remaining sample was reduced to 5443 tweets that we sent for crowdsourcing rating. Finally, after crowdsourcing, our pure sample of signal contained 1642 annotated tweets.

## Results

### Analysis of the Identified Community

The selection described above allowed the identification of users that tweet about their daily lives in the context of HIV. There were 512 unique users that posted the tweets identified as signal. We identified 247/512 (48.2%) male users, 83/512 (16.2%) female, and 182/512 (35.5%) that were unidentified gender. Gender was annotated manually either by first name (if conclusive) or by self-reported identification (if available). About half of users also identified their location. Half of these self-identified locations were in the United States, mainly from New York City and San Francisco. Most of the other locations were from countries with English as the official language, including the United Kingdom, South Africa, and Canada.

The identified community has a large average follower count of about 2300. When looking at the number of within-community followers, for example, the followers of each user who are also in the same community, we find that almost half of the users have no followers within the same community, and several users have almost 100 followers in the community. Furthermore, we studied the friendship relationship between our followers: Are the users that are followed by another member of the community following said follower?. The results indicate that indeed the distribution of these reciprocal relationships is very similar to the one only considering followers, which indicates the strong ties of friendship within our community. Nevertheless, the user friendship network is far from being fully connected, it represents 1516 edges and a structure of 245 subgraphs of connected components.

### Trends in Antiretroviral Drug Tweets

**Figure 2:**
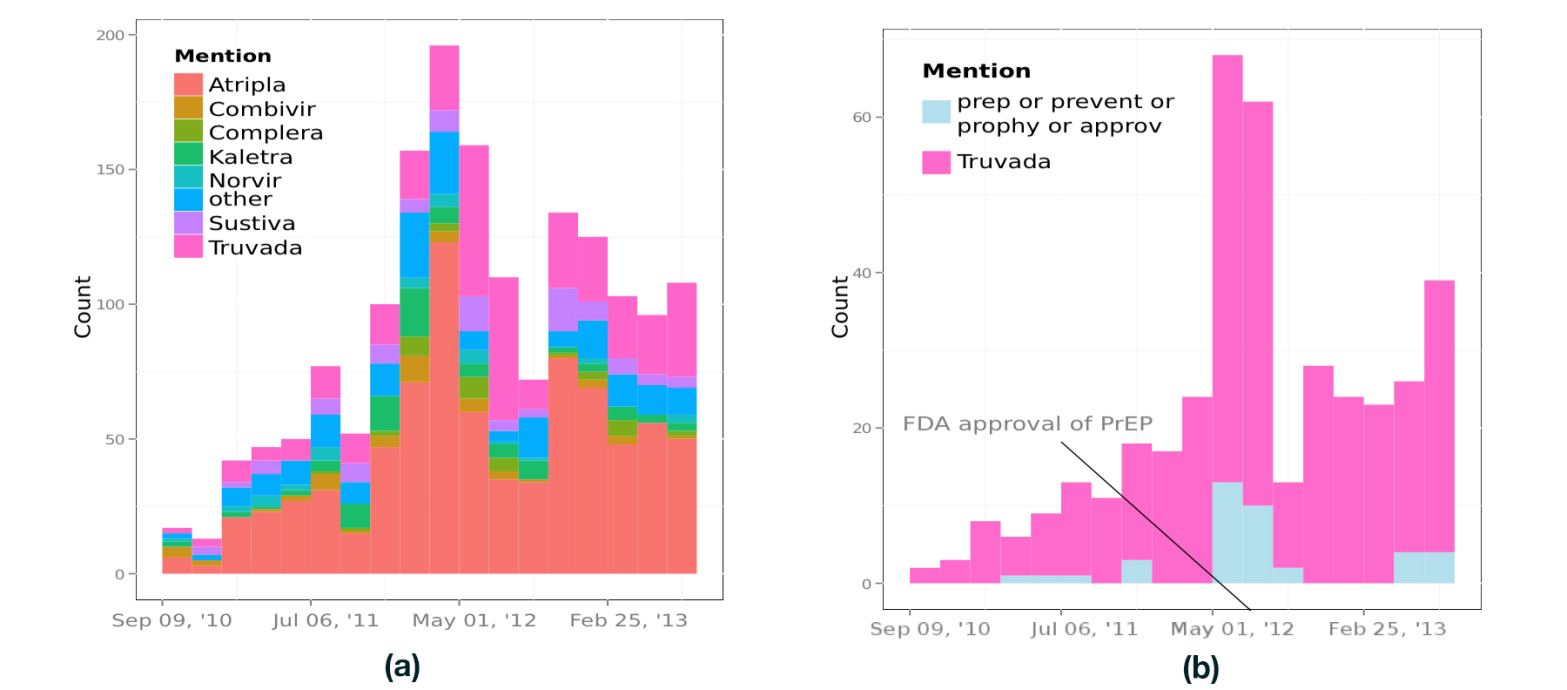
Number of tweets that contain specific mentions as a function of time, from September 2010 until August 2013. Each bin spans a total of 60 days. Panel (a) notes the seven most tweeted drugs separately, and groups the rest of drug mentions under the label "Other". Panel (b) highlights the increase in Truvada tweets at the time of Federal Drug Administration approval of Pre-exposure prophylaxis (PrEP). Specific tokens (on blue) support the association.

Figure 2 shows (left image) a visualization of HIV drugs mentioned by users from September 9, 2010 through August 28, 2013. It shows a peak during the first 6 months of 2012. The total number of tweets is shared among all drugs equally in the first two bins. This trend disappears after the third bin, where Atripla receives more explicit mentions. The only period where Atripla is not ranked first in terms of mentions corresponds to the time spanning from May through August 2012, when Truvada, used as part of a strategy to reduce the risk of HIV infection, gained mentions. We evaluated in more detail the change of ranking of Truvada. Figure 2 displays (right image) the number of Truvada occurrences and the occurrences of four substrings combined: *prep*, *prevent*, *prophy*, and *approv*. The distribution of occurrences of these four tokens aligns with Truvada occurrences, indicating that we captured users tweeting about the approval of Truvada by the US Food and Drug Administration on July 2012 as prophylaxis.

### Reporting on Drug Adverse Effects

**Figure 3:**
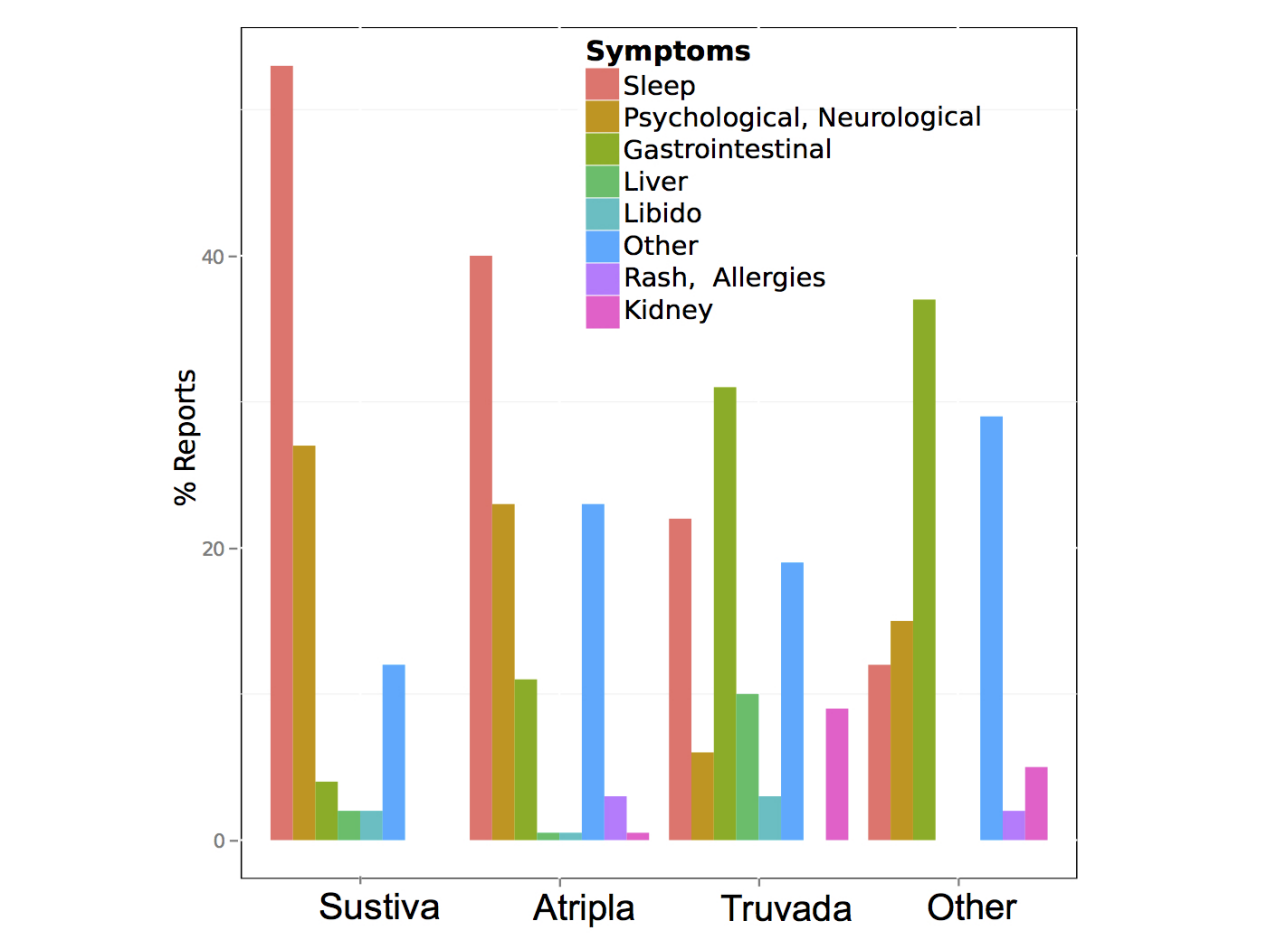
Summary of reported toxicities by unique users on Twitter between September 9, 2010 and August 23, 2013.

Not considering retweets (ie, reposted tweets by other users), 329 out of 353 tweets contained precise information that could be captured as drug adverse effects. Corresponding to the high frequency of use of efavirenz and efavirenz-containing combination treatments (ie, Sustiva, Atripla), most users report problems regarding their sleep, be it a nightmare or a vivid dream, or lack of sleep, as well as symptoms comparable to the effect of psychoactive drugs. These are known adverse effects that may lead to treatment discontinuation [13]. The adverse effects of tenofovir, a component of the commonly used fixed-dose combination pill (Atripla), and of the post exposure prophylaxis regimens (Truvada), is associated with renal toxicity [14] and, in particular in the post exposure setting, with nausea and vomiting [15]. Other drug regimens, such as those including protease inhibitors, are commonly associated with gastrointestinal intolerance. Also, about (27/353) 7.6% of the selected relevant tweets indicate no side effects and that a specific drug was well tolerated. The summary representation of adverse effects attributed to specific drugs or drug combination accurately captures well-recognized toxicities [8,16] (Figure 3 shows this).

### Analysis of User Sentiment

**Figure 4:**
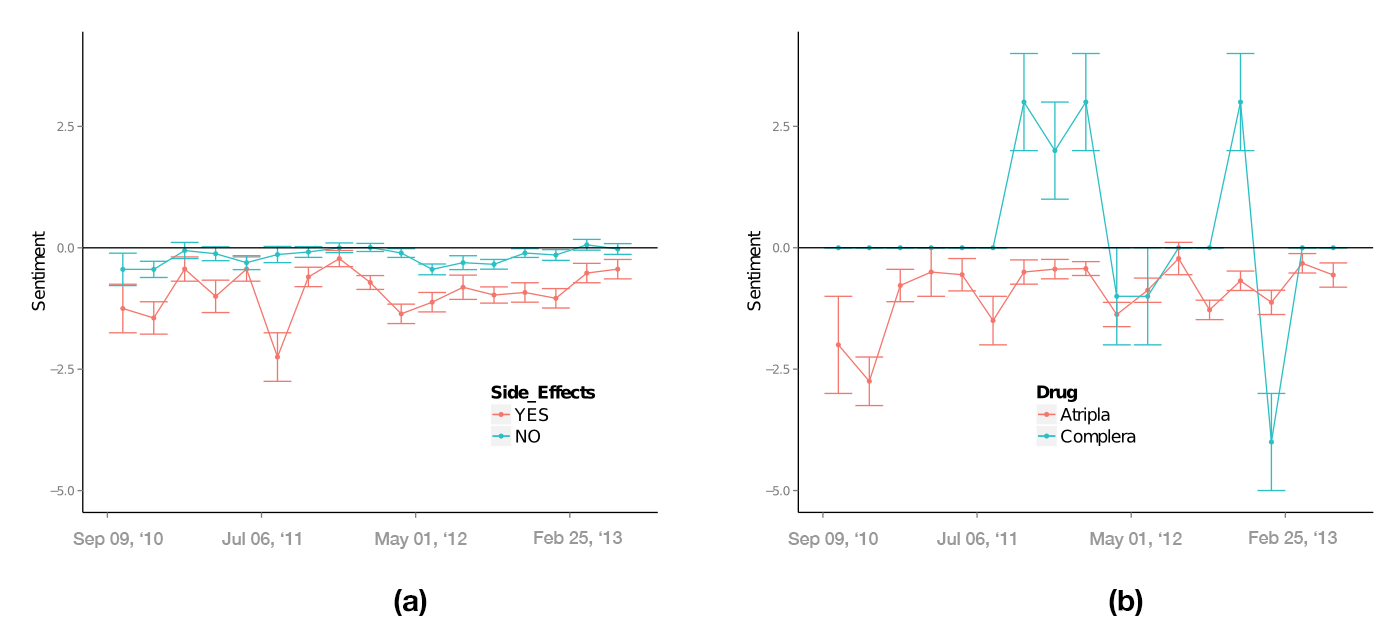
Sentiment score Ψ as a function of time. Panel (a) shows sentiment distributions obtained by considering all tweets and tweets with references to side effects, respectively. Panel (b) depicts sentiment distributions for tweets referring to Atripla and Complera, respectively. The uncertainties are estimated as referred in the Methods section.

Central to this work was the analysis of the observed dynamics of tweets under the concept of sentiment, for example, the expressed emotion associated with the tweet content. Figure 4 shows the computed Ψ over the studied time. We captured an average negative sentiment of -0.178; from a total of 1347 tweets, 348 were rated as negative sentiment, 220 as positive, and 772 as neutral. We also assessed the sentiment associated with tweets specifically referring to adverse effects (Figure 4, left image). From 357 tweets referring to side effects, 238 were associated with negative sentiments, 78 positive, and 37 as neutral. Around May 1, 2012, the number of tweets associating adverse effects peaked at 54, while the average number was 20. This fact was paired with a negative sentiment at that time. Tweet sentiment dynamics appear to differentiate across specific drugs, as illustrated in Figure 4 (right image) with the comparison between Atripla (259 sentiment ratings) and Complera (8 sentiment ratings).

## Discussion

### Using Social Media Data for Public Health

There has been increasing interest on the use of social media data for public health and medicine. Digital epidemiology [1,17] utilizes data from sources such as search engines (Google, Bing, etc), public Internet resources such as Wikipedia [18], and social online networks to track the dynamics of health and disease. Specifically, data from Twitter has been used for the analysis of influenza epidemics [19,20] and influenza vaccine sentiments. More recently, there has been increasing attention to the potential of Internet-based analytics for post marketing assessment of drug toxicity [2,6]. Analysis data from search engines can reconstruct known toxicities based on users queries. Importantly, pharmacovigilance using search query data can expand the spectrum of adverse effects for a given drug or drug interaction on the basis of “guilt by association” [21].

There is little scientific experience in the analysis and use of social media data for HIV. This contrasts with the clear interest that this approach could have for the understanding of global or local epidemiology, for social behavioral studies, and for research on prevention. A study by Young et al [22] served to explore social media for identification of HIV risk through tweets. From more than 550 million tweets, they extracted 9800 geolocated tweets that mapped to areas of high HIV prevalence. On the basis of their study, it has been suggested that an early warning indicator for HIV incidence could emerge by combining HIV prevalence and Twitter data [23].

We aimed here to conduct a first study, to our knowledge, on the possibility that HIV-infected individuals would tweet about their disease, and more specifically, about their experience with drug treatment. It is probable that the specific analysis of drug toxicity, and in particular the possible identification of expanded spectrum of adverse effects, could have been more efficient and successful through the study of queries in search engines such as Google or Bing. However, exploring these questions via analysis of Twitter data offered two unique features: (1) the possibility to capture the emotional context of the tweet, and (2) the estimate of the number of individuals (followers on Twitter) that could be influenced by the content of those tweets.

### Extracting Information From Tweets

Extracting information from tweets requires a significant investment in filtering strategies to separate signal from noise. We placed particular attention on removing tweets that were not written by the identified population (ie, the community of infected individuals). We also went through a number of validation steps that included crowdsourcing and human intervention to assure the validity of filters and support identification of toxicities and hand-rating of sentiments. After applying data cleaning, machine learning, and human rating to an initial dataset of 40 million tweets, we identified 1642 tweets and 512 unique users disclosing personal information in the context of HIV. Although these numbers are not large, individuals that do tweet about their HIV drugs are heavily followed on Twitter, with an average number of followers of almost 2300. Analysis of the target community also revealed strong ties of friendship. This sizable landscape of contacts and possible influence becomes particularly relevant when we examined the sentiment associated with their treatment, as those perceptions can spread broadly to influence the community. As expected, relevant hubs were cities like New York and San Francisco.

The interest of Twitter and of social online networks rests on the fact that information has a personal character that can be captured as “sentiment”. Tweets can be tagged as expressing a positive, negative, or neutral sentiment. About (137/531) 25.8% of tweets on drugs were scored as “negative”. Up to (424/1491) 28.43% of the tweets concerned adverse drug effects, and (281/424) 66.2% of those were scored as negative. It was clear from the data that the concerns expressed in those tweets were accurate as to the nature of the toxicities associated with different components of the anti-HIV combination treatment. For example, the description of adverse effects of efavirenz-containing drugs (Sustiva, Atripla) reconstructs well-established patterns of the neuropsychological toxicity of this compound [8]. The analysis also allowed capturing the rates of reporting, and the associated sentiment in time among those that described side effects. These analyses discriminated across drugs in terms of general acceptance (negative vs neutral-positive sentiments) over time.

The analysis also identified the response of the community to unique events. In Spring 2012, we observed a peak of tweeting about Truvada, at the time that this drug was approved by the Federal Drug Administration for use as post exposure prophylaxis. Although increased media attention would result in increased Twitter communication, we observed that increasingly negative sentiment scores accompanied the increase in tweets, including for tweets referring to adverse effects. This underscores the interest for public health stakeholders promoting new preventive measurements to track these potentially influential signals from online social networks.

The limits of the study are derived from the low numbers of individuals actually reporting on HIV drugs through Twitter. However, as discussed above, the reporting of toxicities is accurate in regards of what is known about those drugs. Different also from search engines, where individuals are actively searching for information possibly related to new adverse events, the content of tweets may simply reflect the awareness of symptoms and signs that have already been announced by health care professionals or in Web resources. The reporting may also reflect preconceived notions of shared beliefs from the community. This observation is in line with the work of Freifeld et al [6] that analyzed 4401 tweets of potential adverse events to 23 medical products and found a *r*=0.75 correlation with consumer-reported Federal Drug Administration Adverse Event Reporting System reports at the System Organ Class level. However, whatever is reported, it indeed reflects on contemporary sentiments among users and their followers. Moreover, previous studies using Twitter were limited by only accessing a small fraction of the tweets. In contrast, we acquired from a specialist service all tweets containing the keywords for the study interval, and we therefore have a full representation of the data for the period of the study. There is also increasing skepticism regarding the use of online social media data as the sole source of information. There has been considerable debate on the recent failure of Google Flu to accurately match influenza trends as compared with the sentinel data from the US Center for Disease Control and Prevention [24]. We believe that our approach that combines automated filters, crowdsourcing, machine learning, and extensive manual validation and scoring may bring more reliable results, even if at the cost of lesser automation. The approach would apply and be a useful addition to real time surveillance of other therapeutic interventions at the population level.

## Appendix

Multimedia Appendix 1 Method used to remove non-English tweets.

## Abbreviations

BDT: Boosted Decision Trees with AdaBoost
HIV: human immunodeficiency virus
SVM: Support Vector Machines

## References

[1] Salathé Marcel, Bengtsson L, Bodnar TJ, Brewer DD, Brownstein JS, Buckee C, Campbell EM, Cattuto C, Khandelwal S, Mabry PL, Vespignani A (2012). Digital epidemiology. PLoS Comput Biol.

[2] Salathé Marcel, Khandelwal Shashank (2011). Assessing vaccination sentiments with online social media: Implications for infectious disease dynamics and control. PLoS Comput Biol.

[3] Cavazos-Rehg P, Krauss M, Grucza R, Bierut L (2014). Characterizing the followers and tweets of a marijuana-focused Twitter handle. J Med Internet Res.

[4] Pagoto S, Schneider KL, Evans M, Waring ME, Appelhans B, Busch AM, Whited MC, Thind H, Ziedonis M (2014). Tweeting it off: Characteristics of adults who tweet about a weight loss attempt. J Am Med Inform Assoc.

[5] Jashinsky J, Burton SH, Hanson CL, West J, Giraud-Carrier C, Barnes MD, Argyle T (2014). Tracking suicide risk factors through Twitter in the US. Crisis.

[6] Freifeld C, Brownstein J, Menone C, Bao Wenjie, Filice Ross, Kass-Hout Taha, Dasgupta Nabarun (2014). Digital drug safety surveillance: Monitoring pharmaceutical products in twitter. Drug Saf.

[7] Sarker A, Ginn R, Nikfarjam A, O'Connor K, Smith K, Jayaraman S, Upadhaya T, Gonzalez G (2015). Utilizing social media data for pharmacovigilance: A review. J Biomed Inform.

[8] Fellay J, Boubaker K, Ledergerber B, Bernasconi E, Furrer H, Battegay M, Hirschel B, Vernazza P, Francioli P, Greub G, Flepp M, Telenti A, Swiss HIV Cohort Study (2001). Prevalence of adverse events associated with potent antiretroviral treatment: Swiss HIV Cohort Study. Lancet.

[9] Keiser O, Fellay J, Opravil M, Hirsch H, Hirschel B, Bernasconi Enos, Vernazza Pietro L, Rickenbach Martin, Telenti Amalio, Furrer Hansjakob, Swiss HIV Cohort Study (2007). Adverse events to antiretrovirals in the Swiss HIV Cohort Study: Effect on mortality and treatment modification. Antivir Ther.

[10] Sadilek A, Brennan S, Kautz H, Silenzio V (2013). nEmesis: Which restaurants should you avoid today?.

[11] Günthard Huldrych F, Aberg JA, Eron JJ, Hoy JF, Telenti A, Benson CA, Burger DM, Cahn P, Gallant JE, Glesby MJ, Reiss P, Saag MS, Thomas DL, Jacobsen DM, Volberding PA, International Antiviral Society-USA Panel (2014). Antiretroviral treatment of adult HIV infection: 2014 recommendations of the International Antiviral Society-USA Panel. JAMA.

[12] Hoecker A, Speckmayer P, Stelzer J, Therhaag J, Toerne VE (2007). TMVA - Toolkit for Multivariate Data Analysis.

[13] Ford N, Shubber Z, Pozniak A, Vitoria M, Doherty M, Kirby C, Calmy A (2015). Comparative safety and neuropsychiatric adverse events associated with efavirenz use in first-line antiretroviral therapy: A systematic review and meta-analysis of randomized trials. J Acquir Immune Defic Syndr.

[14] Ryom L, Mocroft A, Lundgren JD (2014). Antiretroviral therapy, immune suppression and renal impairment in HIV-positive persons. Curr Opin HIV AIDS.

[15] Coutinho B, Prasad Ramakrishna (2013). Emtricitabine/tenofovir (Truvada) for HIV prophylaxis. Am Fam Physician.

[16] Fagard C, Le BM, Günthard Huldrych, Hirsch HH, Egger M, Vernazza P, Bernasconi E, Telenti A, Ebnöther Corinna, Oxenius A, Perneger T, Perrin L, Hirschel B, Swiss HIV Cohort Study (2003). A controlled trial of granulocyte macrophage-colony stimulating factor during interruption of HAART. AIDS.

[17] Salathé Marcel, Freifeld CC, Mekaru SR, Tomasulo AF, Brownstein JS (2013). Influenza A (H7N9) and the importance of digital epidemiology. N Engl J Med.

[18] McIver DJ, Brownstein JS (2014). Wikipedia usage estimates prevalence of influenza-like illness in the United States in near real-time. PLoS Comput Biol.

[19] Chew C, Eysenbach G (2010). Pandemics in the age of Twitter: Content analysis of Tweets during the 2009 H1N1 outbreak. PLoS One.

[20] Kim E, Seok JH, Oh JS, Lee HW, Kim KH (2013). Use of hangeul twitter to track and predict human influenza infection. PLoS One.

[21] White R, Tatonetti N, Shah N, Altman R, Horvitz Eric (2013). Web-scale pharmacovigilance: Listening to signals from the crowd. J Am Med Inform Assoc.

[22] Young SD, Rivers C, Lewis B (2014). Methods of using real-time social media technologies for detection and remote monitoring of HIV outcomes. Prev Med.

[23] Stoové Mark A, Pedrana Alisa E (2014). Making the most of a brave new world: Opportunities and considerations for using Twitter as a public health monitoring tool. Prev Med.

[24] Lazer D, Kennedy R, King G, Vespignani A (2014). Big data. The parable of Google Flu: Traps in big data analysis. Science.

