# Supplementary material for: Identifying Adverse Effects of HIV Drug Treatment and Associated Sentiments Using Twitter
Source: JMIR Public Health Surveill. 2015 Jul 27;1(2):e7. doi: 10.2196/publichealth.4488 (PMC4869211; doi:10.2196/publichealth.4488)
Supplement: Multimedia Appendix 1 [file publichealth_v1i2e7_app1.pdf]

APPENDIX AND SUPPLEMENTARY FIGURES AND TABLES

Figure S1

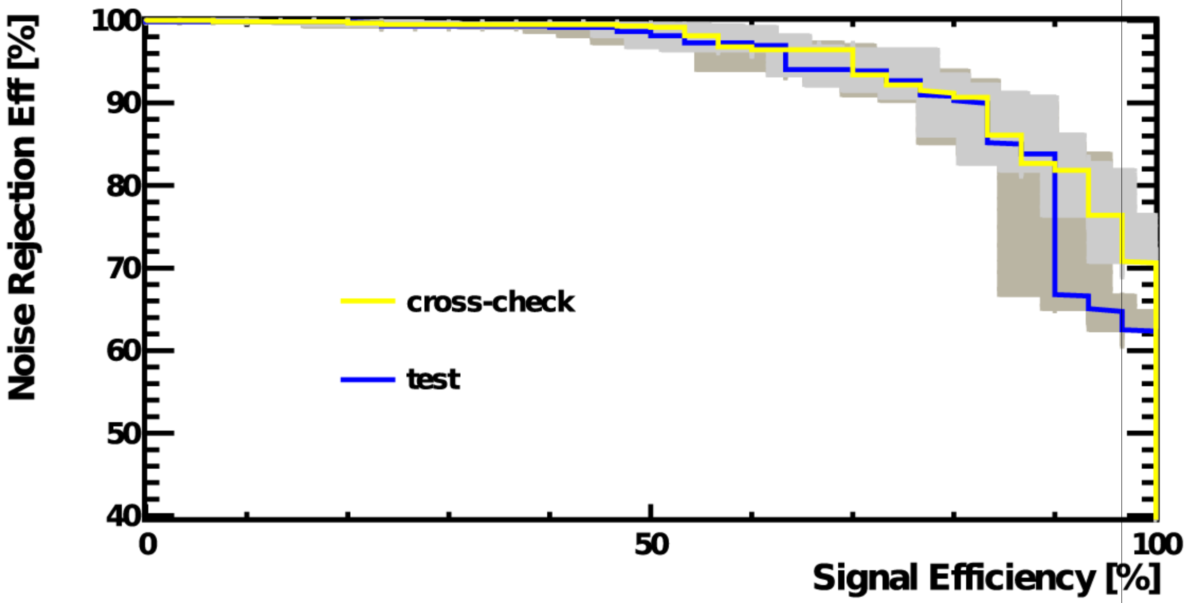

Classifier efficiency on targeted tweets (Signal) versus noise rejection efficiency for two different testing samples. Gray bands represent statistical uncertainties.

Figure S2

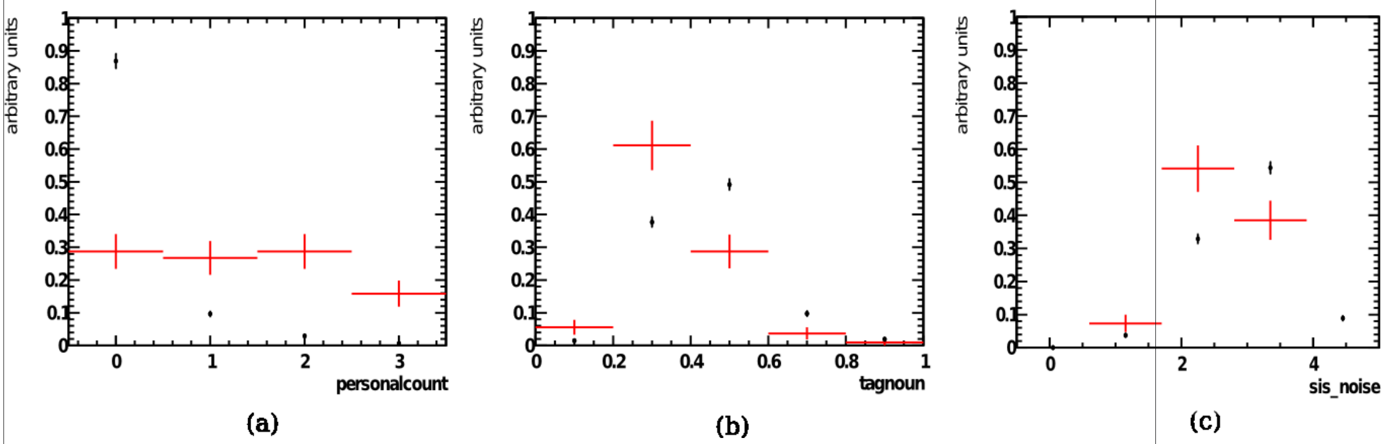

Three of the features obtained for signal (red) and noise (black). The statistical uncertainties are represented on the vertical axis. These figures have been obtained with 109 signal events and 1,809 noise events.

Figure S3

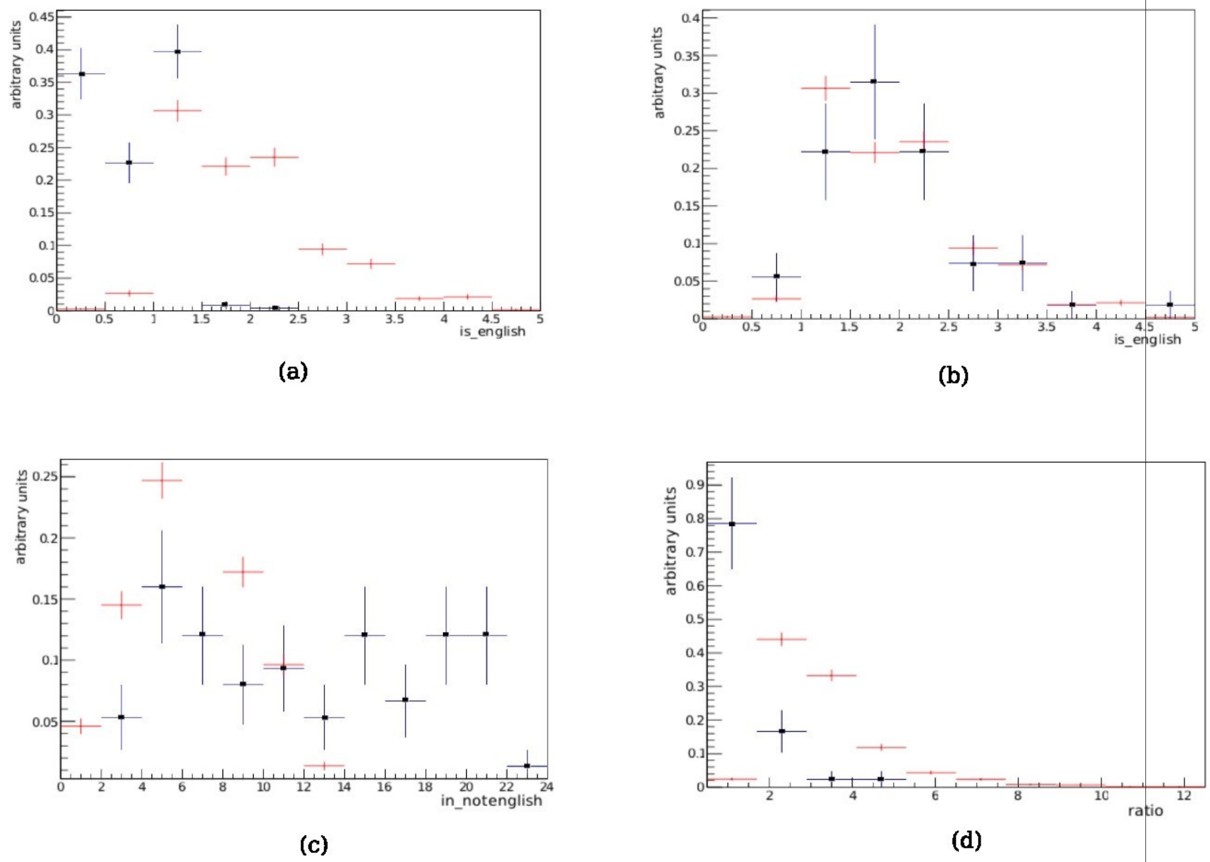

Foreign Language Removal figures. Panel (a) shows distributions of is english for tweets annotated as non-English (blue with black dots) and the rest of tweets (red). Panel (b) shows distributions of is english for signal (blue with black dots) and tweets annotated as English (red). Panel (c) shows distributions of in_notenglish for tweets annotated as non-English (blue with black dots) and the rest of tweets (red). Panel (d) shows (wordcount / in_notenglish) for tweets annotated as non-English (blue with black dots) and the rest of annotated tweets (red).

Supplementary Table 1

Most common tokens (left column) in the sample of 316,081 tweets. Total number of appearances (right column) and number of tweets it appears (middle column).

Token	Tweets	Total
hiv	199159	227374
t.co	197078	202777
drug	94680	104102
rt	79181	85599
treatment	69529	73126
truvada	44325	49363
anti	44591	45885
onlin	27892	32538
buy	27363	30218
anti-hiv	29583	29900
prevent	28018	29151
new	26263	27667
fda	25850	27620
approv	24507	26773
retrovir	21734	25647
aid	23863	24804
bit.ly	22462	22932
news	16812	18168
generic	14851	17865
de	12959	17314

Supplementary Table 2

Yields before and after requirements.

Type	Yield Before	Yield After
Signal	94	88
Noise (all)	2717	2179

Noise (non-English)	472	29

Feature extraction

Hereafter we give the definition of all features extracted from each tweet:

- modalcount: number of times the words ”should”, ”shoulda”, ”can”, ”could”, ”may”, ”might”, ”must”, ”ought”, ”shall”, ”would”, and ”woulda” occur in the tweet;
- futurecount: number of times the words ”going”, ”will”, ”gonna”, ”should”, ”shoulda”, ”ll”, ”d” occur in the tweet;
- personalcount: number of times the words ”i”, ”me”, ”my”, ”mine”, ”ill”, ”im”, ”id”, ”myself” occur in the tweet;
- negative: number of times the words ”not”, ”wont”, ”nt”, ”shouldnt”, ”couldnt” occur in the tweet;
- secondpron: number of times the words ”you”, ”youll”, ”yours”, ”yourself” occur in the tweet;
- thirdpron: number of times the words ”he”, ”she”, ”it”, ”his”, ”her”, ”its”, ”himself”, ”him”, ”herself”, ”itself”, ”they”, ”their”, ”them”, ”themselves” occur in the tweet;
- relatpron: number of times the words ”that”, ”which”, ”who”, ”whose”, ”whichever”, ”whoever”, ”whoever” occur in the tweet;

- dempron: number of times the words ”this”, ”these”, ”that”, ”those” occur in the tweet;
- indpron: number of times the words ”anybody”, ”anyone”, ”anything”, ”each”, ”either”, ”everyone”, ”everything”, ”neither”, ”nobody”, ”somebody”, ”something”, ”both”, ”few”, ”many”, ”several”, ”all”, ”any”, ”most”, ”none”, ”some” occur in the tweet;
- intpron: number of times the words ”what”, ”who”, ”which”, ”whom”, ”whose” occur in the tweet;
- percent: number of % symbols in the tweet;
- posnoise: number of times the words ”new”, ”pill”, ”state”, ”states”, ”stats”, ”drug”, ”people”, ”approved”, ”approve”, ”approves”, ”approval”, ”approach”, ”prevention”, ”prevent”, ”prevents”, ”prevented” occur in the tweet;
- pharmacy: number of times the words ”cvs”, ”hospital”, ”pharmacy”, ”doctor”, ”walgreens”, ”target”, ”clinic”, ”meds”, ”medication”, ”medications” occur in the tweet;
- is_notenglish: number of times words contained in a list of words extracted from annotated tweets as not English occur in the tweet;
- regularpast: number of words ending with ed contained in the tweet;
- gerund: number of words ending with ing contained in the tweet;
- nment: number of words ending with ment contained in the tweet;
- nfull: number of words ending with full contained in the tweet;

- tagadj: ratio of the number of adjectives tagged using NLTK [1] in the tweet by the total number of words in the tweet;
- tagverb: ratio of the number of verbs tagged using NLTK [1] in the tweet by the total number of words in the tweet;
- tagprep: ratio of the number of prepositions tagged using NLTK [1] in the tweet by the total number of words in the tweet;
- tagnoun: ratio of the number of nouns tagged using NLTK [1] in the tweet by the total number of words in the tweet;
- tagconj: ratio of the number of conjunctions tagged using NLTK [1] in the tweet by the total number of words in the tweet;
- tagadv: ratio of the number of adverbs tagged using NLTK [1] in the tweet by the total number of words in the tweet;
- tagto: ratio of the number of to tagged using NLTK [1] in the tweet by the total number of words in the tweet;
- tagdeterm: ratio of the number of determinants tagged using NLTK [1] in the tweet by the total number of words in the tweet;
- sis_noise: ratio of the similarity of the tweet with a corpus of annotated noise tweets by its uncertainty. To compute the similarity we first create a sparsity matrix of the tokens in the annotated corpus, then count the number of times the token appears in the tweet and divide by the number of elements in the corpus. We use scikit-learn [2] library in several parts of the definition of sis noise;
- sis_signal: ratio of the similarity of the tweet with a corpus of annotated noise tweets by its uncertainty. To compute the similarity we first create a

sparsity matrix of the tokens in the annotated corpus, then count the number of times the token appears in the tweet and divide by the number of elements in the corpus. We use scikit-learn [2] library in several parts of the definition of sis noise;

- in_english: number of words in corpus of English words [1] divided by number of words in corpuses of Spanish, Portuguese, French, German, Dutch, Italian, Russian, Swedish, and Danish [1]. We add one in both numerator and denominator to avoid dividing by zero.
- bigrams noise: number of bigrams found in tweet that are contained in list of bigrams of noise annotated bigrams corpuses divided by the total number of bigrams from annotated corpuses;
- bigrams signal: number of bigrams found in tweet that are contained in list of bigrams of signal annotated bigrams corpuses divided by the total number of bigrams from annotated corpuses;
- isolation: number of keywords contained in tweet minus one;
- common_noise: sum of the weights of each word contained in most common 25% of words in noise annotated tweets;
- common_signal: sum of the weights of each word contained in most common 25% of words in signal annotated tweets;
- wordscount: number of words in tweet;
- tweetlength: number of characters in tweet.

Foreign Language Removal

We extracted features from tweets to be able to feed a machine learning algorithm, and separate noise from signal more efficiently. Nevertheless, even if we had the best semantic features, the machinery would have difficulties in separating tweets that are non-English. In this section we propose a method to suppress almost all of foreign tweets without losing much signal.

We recall that we used tweets rated as non-English as our control sample. The distributions of in_english for tweets rated as non-English and the rest are shown in the Figures and Tables section. 60% of non-English tweets and 2.5% of the rest remain at values of in_english below 1. Therefore, we would reject 2.5% of signal tweets and 60% of non-English tweets if we required in_english≥1. The assumption that the distributions of is english for signal tweets and English tweets are identical is validated by Panel (b). Almost all values of in_notenglish are equal to wordscount for tweets annotated as non-English. Also, foreign language encryption leads to tweets with more than 150 characters. Overall, the requirements: in_english ≥ 1 and tweetlength < 150 and in_notenglish < 14 and (wordscount / in_notenglish) > 1 lead to an estimated 6% signal loss, while removing 20% of all noise and 94% of non-English tweets.

REFERENCES

1. Bird, Steven, Edward Loper and Ewan Klein (2009). Natural Language Processing with Python. O'Reilly Media Inc.

2. Pedregosa, F. and Varoquaux, G. and Gramfort, A. and Michel, V. and Thirion, B. and Grisel, O. and Blondel, M. and Prettenhofer, P. and Weiss, R. and Dubourg, V. and Vanderplas, J. and Passos, A. and Cournapeau, D. and Brucher, M. and Perrot, M. and Duchesnay, E., "Scikit-learn: Machine Learning in Python", Journal of Machine Learning Research, 2011
